# Population structure of *Phanaeus vindex* (Coleoptera: Scarabaeidae) in SE Michigan

**DOI:** 10.1093/jisesa/iead050

**Published:** 2023-07-03

**Authors:** Thomas Wassmer, Elise Armstrong

**Affiliations:** Department of Biology, Siena Heights University, 1247 E. Siena Heights Drive, Adrian, MI 49221, USA; Department of Biology, Siena Heights University, 1247 E. Siena Heights Drive, Adrian, MI 49221, USA

**Keywords:** mark release recapture, capture–mark–recapture, dung beetle, population size, dispersal

## Abstract

Until now, little is known about the population structure and mobility of temperate dung beetles including the rainbow scarab, *Phanaeus vindex* (MacLeay 1819), although this knowledge is essential for their conservation as pastures become increasingly rare and the landscape fragmented by monocultures and urbanization. Here, we estimated population size, longevity, and dispersal within and between pastures. For 3 yr, we life-trapped beetles every week on 2 adjacent farms in SE Michigan, determined their sex, male morph, and size, and marked their elytra with individual tattoo patterns before releasing them. We marked a total of 470 rainbow scarabs of which 14 were recaptured once and 2 were recaptured twice. The sex ratio was not significantly sex-biased but fluctuated between months with no apparent uniformity between years. While the minor to major male ratios were unbiased in 2019 and 2020, they were marginally minor-biased in 2021. The gross population estimates for the 2 farms were 458–491 and 217 rainbow scarabs, respectively. Beetles traveled distances of up to 178 m within farms. No beetles dispersed between farms. One large female was recaptured after 338 days documenting the first cold hardiness and long lifespan of a cold-temperate dung beetle species in the wild. The low population estimates on both farms indicate 2 vulnerable populations with no or extremely limited connectivity. Supplementary funding for the land stewardship of small-scale cattle farmers could stabilize populations of native dung beetles and maintain their ecosystem services.

## Introduction

Large paracoprid dung beetles (Doube 1990) provide essential ecosystem services (Nichols et al. 2008, Stanbrook and King 2022, deCastro-Arrazola et al. 2023). First, they break up and remove animal waste (excrement) from the soil surface into their subterraneous galleries and brood chambers (Doube 1990, Sladecek et al. 2013) and by doing so, enhance soil microbial communities (Slade, et al. 2016, Slade et al. 2017), which accelerate the mineralization of nutrients (Haynes and Williams 1993, Maldonado et al. 2019) and their uptake into plants (Yamada et al. 2007). Secondly, by removing dung pats, they remove habitats in which intestinal worms (Sands and Wall 2017), ectoparasites, and disease vectors (Ridsdill-Smith and Hayles 1990, Nichols et al. 2008) would otherwise flourish. Lastly, they also reduce the development of greenhouse gases that would be generated if dung pats are decomposing above ground (Slade et al. 2016b, Verdú et al. 2020).

Despite their ecological importance, little is known about the populations of large paracoprid dung beetles. This is most likely due to the difficulties in marking beetles reliably to conduct mark–release–recapture (MRR) studies also known as capture–mark–recapture (CMR) studies (Krebs 1999, Seber 2002, Henderson 2021). Despite these obstacles, the knowledge gap on dung beetle populations is astonishing as populations play a central role in the assembly of ecological communities, ecosystems, and biodiversity, and determine the resilience or vulnerability of other species. Besides the studies by Hanski (1980) and Roslin (2000) all MRR studies on dung beetles so far were conducted in tropical Meso- and South America (e.g., Escobar and Chacón de Ulloa 2000, Arellano et al. 2008, Cultid-Medina et al. 2015, 2015, da Silva and Hernández 2015, Barretto et al. 2019).

The rainbow scarab, *Phanaeus vindex* Macleay 1819, is a beautifully colored, large paracoprid dung beetle native to the Southern United States reaching its northern range limit in SE Michigan (Dickey 2007). While many aspects of its biology are researched (Halffter et al. 1974, Halffter and Lopez 1977, Edmonds 1994, Rasmussen 1994, Dickey 2007, Price and L. May 2009, Paris et al. 2013, Kirkpatrick and Sheldon 2022), almost nothing is known about its populations. The purpose of this study was to use an MMR strategy to determine several vital population metrics such as size, sex ratio, ratio between male major and minor morphs, longevity, and dispersal. Such data are needed more than ever as the populations of the rainbow scarab like many other native dung beetle populations are in peril due to habitat loss and fragmentation leading to loss of connectivity (Cardoso et al. 2020, Watling et al. 2020), and loss of reliable food sources (Foster et al. 2020) driven by the intensification of agriculture (Blayney 2004, USDA 2016, Clay et al. 2020) and urbanization (Liu et al. 2016, Simkin et al. 2022). The resulting extinction pressure is further accelerated by climate change (Tocco et al. 2021) and the displacement of native by invasive dung beetle species (Pokhrel et al. 2020).

## Materials and Methods

### Location

**Fig. 1.**
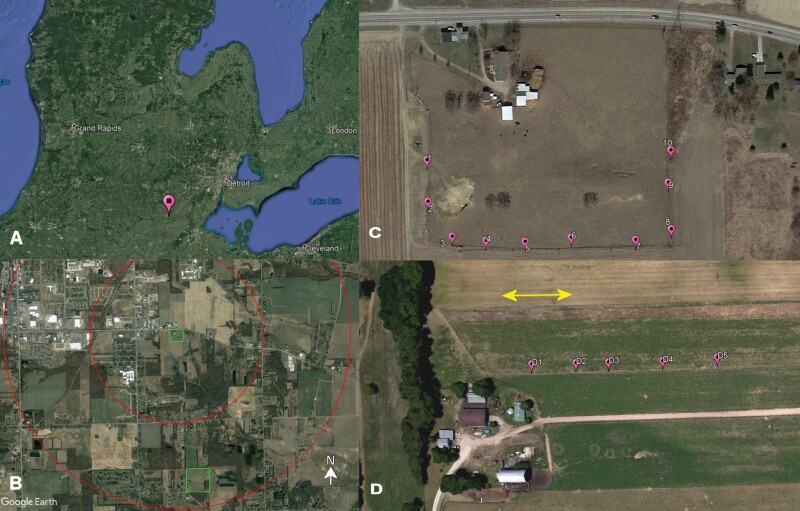
(A) Study location in SE Michigan (pin), (B) location of Carpenter farm (green rectangle in center) and Deline Farm (green rectangle on bottom) within the local landscape mosaic of 6 by 4 km. The concentric circles denominate 1.5 and 3 km around Carpenter Farm. (C) Trap array (pins) along the fence line of Carpenter Farm and D between permanent paddocks on Deline Farm. The arrow indicates the average distance of 50 m between traps. Maps generated using Google Earth Pro.

We collected rainbow scarabs on the 4.9 ha Carpenter Farm (CF, 41.874°, −84.010°, elevation 243 m above sea level) during all 3 yr of this study (2019–2021). In 2021, we also collected on the 16.3 ha Deline Farm (DF, 41.852°, −84.006°, elevation 234 m above sea level), which is the only other pasture within a 3-km radius (straight distance approx. 2.4 km, Fig. 1B). Both farms are in SE Michigan and are embedded in a landscape mosaic dominated by urbanization and row crops (Fig. 1). Carpenter Farm harbored about 45 cattle of Black Angus and Hereford-Angus breeds, which were moved to a more productive pasture from mid spring to late autumn during each year of the study and were replaced by a small herd of goats (Wassmer 2020b). No growth promoters, or antibiotics were used on a regular basis. Pumpkins were fed to the cattle in the fall as a natural dewormer. The soil types of Carpenter Farm are St. Clair loams (fine, illitic, mesic oxyaquic Hapludalfs) and Plainfield and Ottawa loamy sands (mixed, mesic typic Udipsamments) (SoilWeb-Earth 2022). Forage species found on the pasture are mainly *Lolium spec.* (L.) (Poales: Poaceae), *Festuca arundinacea* (Schreb.) (Poales: Poeaceae), and native grasses. Deline Farm is on Plainfield loamy sands (excessively drained Udipsamments), Berrien sandy loam (moderately drained Udipsamments), and Granby loamy sand soils (poorly drained endoaquolls) (SoilWeb-Earth 2022) and the year-round home to 45–50 heads of Hereford cattle. No growth promoters, and antibiotics were used on a regular basis. Dewormers were used twice a year. The grassland is dominated by cool-season grasses and Fabaceae. Long-term climate data (Weatherbase 2022) for Adrian, MI (3 and 5 km NW of the sampling sites, respectively) identify average high and low temperatures in the coldest months (January and February) of 0 and −8 °C, respectively. Average high and low temperatures in the hottest month (July) are 29 and 15 °C, respectively. Average annual precipitation is 925 mm. We spaced 5–10 traps approximately 50 m apart from each other along a fence line around or between pasture paddocks (Fig. 1C and D).

### Trap Setup

**Fig. 2.**
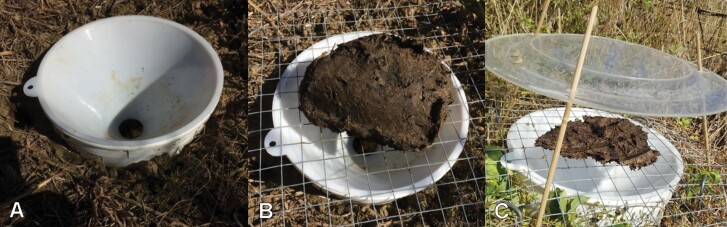
Live trap design. (A) 1-Gallon bucket in the ground inserted funnel. (B): 19 mm wire mesh placed over the funnel and staked down with dung bait. (C) Transparent rain guard.

Beetles were captured alive in pitfall traps with an 8-in diameter × 6-inch height plastic funnel inserted into a buried 1 Gallon bucket (Fig. 2), which allows beetles to fall into the pit but makes it difficult for them to get out. We baited the traps with approximately 400 g of freshly collected cow dung placed on a 19 mm wire mesh to exclude vertebrates and used a rain guard to protect the trap from flooding. In 2019 and 2020, we positioned 10 evenly spaced traps along the fenced perimeter of CF (Fig. 1C). In July 2021, we reduced the number of traps on CF to the 5 traps along the southern end of the pasture and added 5 evenly spaced traps to a permanent paddock fence within the neighboring DF (Fig. 1D). The placement of traps below or just outside of fence lines avoided damages to the traps by farm animals. Traps were spaced approximately 50 m apart to allow for independent sampling (Larsen et al. 2008). On CF in 2019, we trapped weekly between 9 June and 6 September, in 2020 between 7 May and 4 October, and in 2021 between 4 May and 10 October. DF was only sampled in 2021 between 3 July and 10 October. On every trapping day, we set traps between 8 and 10 am and collected beetles for 24 h.

### Data Collection

**Fig. 3.**
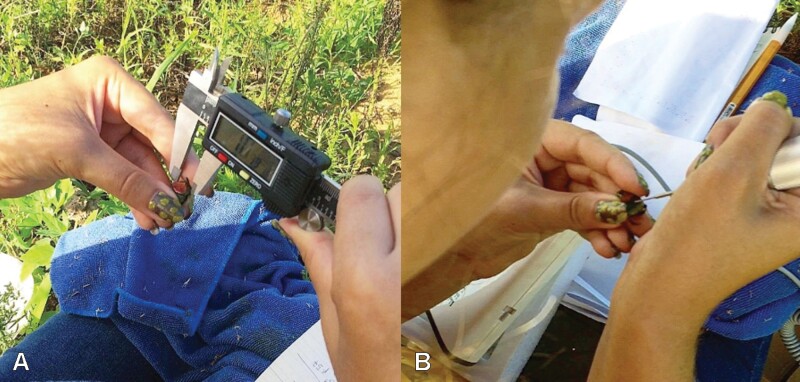
Measuring and marking beetles in the field. (A) Measuring of a rainbow scarab’s pronotal width using a digital caliper. (B) Applying a unique dot pattern tattoo onto a rainbow scarab’s elytra. A video of the process is provided as Supplementary Materials.

**Fig. 4.**
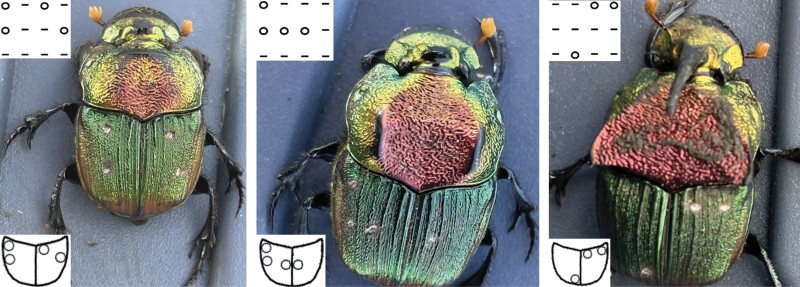
Three examples of individual tattoo markings with the software generated patterns on the inserts in the upper left corners of each beetle photograph, the idealized patterns on the insert at the lower left corner and the actual drill pattern shown in the beetle photographs. Also illustrated is the clear sexual dimorphism, showing a female on the left and 2 males in the middle and to the right with a minor male in the center and a major male in the right panel.

Each living rainbow scarab was restrained between index finger and thumb (Fig. 3A), sexed, and males were categorized as minor or major morphs (Fig. 4). Then we measured the length of each captured rainbow scarabs from clypeus to pygidium and the width of the pronotum using a 0–150 mm digital caliper (Vinca DCLA-0605, Clockwise Tools Inc., Valencia, CA, Fig. 3A). Between 25 June 2021 and 26 September 2021 we also measured the horn length of male rainbow scarabs from the base to the tip of the horn.

To investigate the population structure and dispersal, we followed a Capture-Mark-Recapture (CMR) approach also known as Mark-Release-Recapture (MRR) studies. In most of 2019 (until 23 August 2019), beetles were individually marked right at the capture site by using medium point oil-based paint markers (Uni-Paint PX-20, Sanford L.P., Oak Brook, Illinois, USA) to apply a pattern of white, yellow, or red dots to the beetle’s elytra. Due to a low recapture rate, we shifted the marking technique from 30 August 2019 on to a dot pattern tattooed to the beetles’ elytra using a portable nail drill (AIRSEE NS2036, Rechargeable 30000RPM Electric Nail Drill Professional, airsee.us) with 0.5–0.9 mm Tungsten Vanadium steel drills (Esslinger and Company, Saint Paul MN) (Figs. 3B and 4). No leakage of hemolymph or any other damage was observed after the marking procedure. A video of the drilling procedure was uploaded to Supplementary Materials (Supplementary Video S1). Due to the change in marking technique in 2019, we based our population estimates only on the mark recapture records for 2020 and 2021.

The dot patterns for the tattoo markings (Fig. 4) were generated from all possible permutations of 1–6 dots on each elytron with the help of a Visible Basic program written by Roberto Refinetti in 2019 that was based on an underlying algorithm developed by Andrew-David Bjork. After marking, we took a picture of each beetle and released them next to the trap they were captured in.

### Data Analysis

#### Population estimates.

Sex ratio was calculated as male/(male + female). This results in a sex ratio of 0.5 if the number of males and females is equal (Wilson and Hardy 2002). This method is the most recommended for estimating sex ratios in nature given that it considers individuals as discrete units and thereby reflects the relative abundance of each sex in a population (Ancona et al. 2017). As the expected sex ratio is 0.5 (1:1), we used chi-square (χ^2^) tests to determine if the observed sex ratio was statistically different from the expected ratio. The same rational and procedures were applied to the ratio between major and minor morphs in the male sex.

The observed lifespan was calculated as the number of days between the first capture and last recapture. The proportion of beetles recaptured throughout the sampling period was determined by dividing the number of recaptures by the total number of captures. MRR/CMR data were analyzed using the MARK 9.0 and 8.2 software (White and Burnham 1999, White and Cooch 2019), under the assumptions of the open population Jolly–Seber model with the POPAN parametrization (Schwarz and Arnason 1996) to estimate the population size *N* and 3 primary parameters: *φ*_*i*_—apparent survival probability, *p*_*i*_—recapture probability (catchability), and *pent*_*i*_—the probability of entering the population (recruitment rate: combining birth and immigration). Given the low recapture rate in this study, the only model we considered *a priori* was *φ*(.) *p*(.) *pent*(t), in which we kept survival *φ* and catchability *p* constant *φ*(.) *p*(.) because we could not estimate a time variation in these variables whereas we could for the recruitment rate *pent*(t) as we captured many new individuals in each sampling event. We are aware that survival and catchability may in fact not be constant through time which may lead to biased population size estimates through time (N. Schtickzelle, personal communication).

#### Dispersal distances and patterns.

Straight line distances between the traps that captured individual beetles were summed up to obtain a conservative measure of the overall movements for each beetle captured more than once. Analyses were carried out by pooling distance values obtained from the 2 farms. The Mann–Whitney *U*-test was used to test differences between distances covered by males and females.

## Results

### Morphometrics

**Fig. 5.**
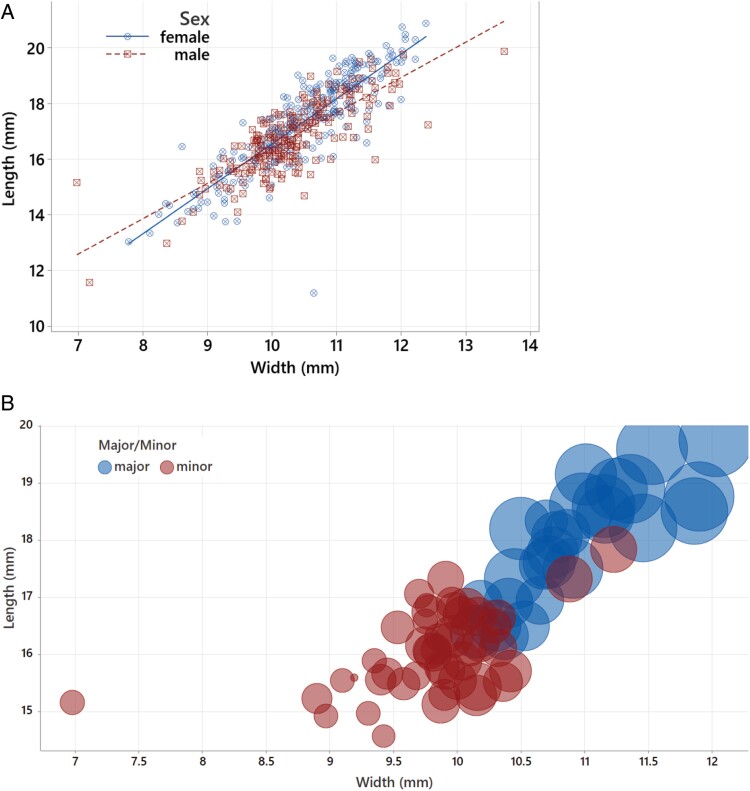
A) Scatterplot of the lengths and widths of 458 rainbow scarabs. Females shown in blue circles, males shown in red squares. The distributions for both sexes overlap but females are on average longer and wider. B) Bubble plot of 74 male rainbow scarabs for which horn length was recorded. Minor males shown in red – major males in blue. The position of the bubbles shows the lengths and widths of the beetles while the bubble diameter is proportional to the horn length.

Length and width of rainbow scarabs are well correlated (*r* = 0.834, *P* < 0.0001). For both sexes, both parameters are normally distributed, and the distributions overlap each other (Fig. 5A) with females being on average slightly larger and wider than males (Table 1). Both differences are significant (length: *F*_(1,456)_ = 16.83, *P* < 0.0001, width: *F*_(1,456)_ = 5.69, *P* = 0.017).

**Table 1.** Morphometrics of rainbow scarabs *Phanaeus vindex*

Sex/morph	Number	Length	Width	Horn length
Female	241	17.350 ± 1.644	10.500 ± 0.873	—
Male	217	16.781 ± 1.277	10.312 ± 0.804	3.149 ± 2.301
Minor	125	16.117 ± 1.032	9.865 ± 0.615	1.727 ± 0.765
Major	92	17.683 ± 0.994	10.920 ± 0.610	5.624 ± 1.951

Male minor and major morphs are well separated from each other by width and length measurements as well as horn length (Table 1, Fig. 5B). Both length and width are well correlated to horn length (*r* = 0.836 and 0.833, *P* < 0.001), and length, width, and horn length are all significantly larger in major males as compared to minor males. Male rainbow scarabs showed significant changes over the sample months in the average length (*F*_(4,212)_ = 3.55, *P* = 0.008) but not width (*F*_(4,212)_ = 0.85, *P* = 0.494). A Tukey post hoc test identified that male beetles were significantly longer in June as compared to August (17.263 ± 1.226 vs. 16.477 ± 1.224, *P* < 0.05). Female beetles did not show any significant seasonal changes in width or length.

### Recapture Rate

Over the 3 yr of the study, we conducted 59 weekly mark-recapture sessions of which 11 did not capture any rainbow scarabs: 2 in September 2019, 2 in May 2020, 1 in mid-July 2020, 2 in late September and early October 2020, 2 in May 2021 and 2 in October 2021. During the 48 mark-recapture sessions that yielded rainbow scarabs, we captured 488 rainbow scarabs alive consisting of 470 individual beetles of which 15 were recaptured once (2 of them on the same day they were marked but in a different trap) and 2 were recaptured twice resulting in an overall recapture rate (single recapture, same day excluded) of 3.19%. Table 2 provides the capture and recapture numbers and recapture rates for each of the 3 yr of this study and both farms sampled in 2021.

**Table 2.** Capture and recapture numbers, and recapture rate (per year and farm, single recapture, same day excluded)

Farm	2019	2020	2021	2021	Sum 2021	Total
	CF	CF	CF	DF		
N of traps	10	10	10 until 27 June then 5	5 from 3 July on	10	
*N* individual beetles captured	106	133	148	83	231	470
*N* recaptured once (*N* same day)	2	5 (1)	4 (1)	3	7 (1)	14 (2)
*N* recaptured twice	0	1	1	0	1	2
Recapture rate (%)	1.89	3.76	2.70	3.62	3.03	2.98

### Longevity and Overwintering

**Fig. 6.**
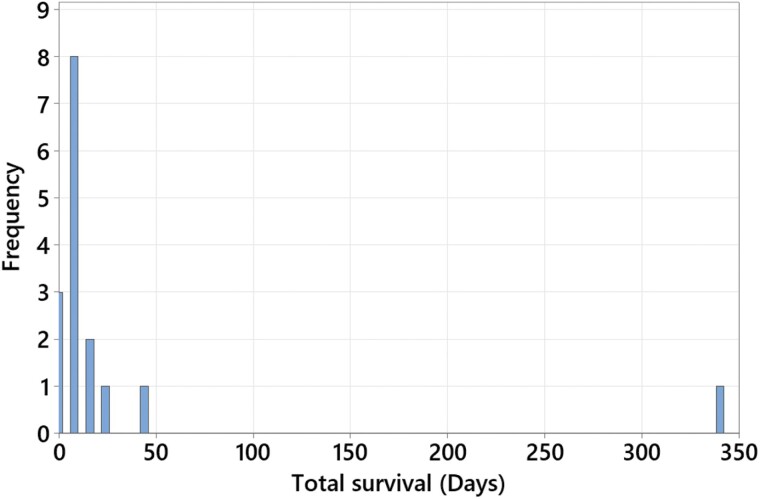
Histogram of the time intervals between the marking and the final rapture event (minimum survival/longevity) of all 16 recaptures (same day recaptures included). Bin width 4 days, labels are centered on the bins.

**Fig. 7.**
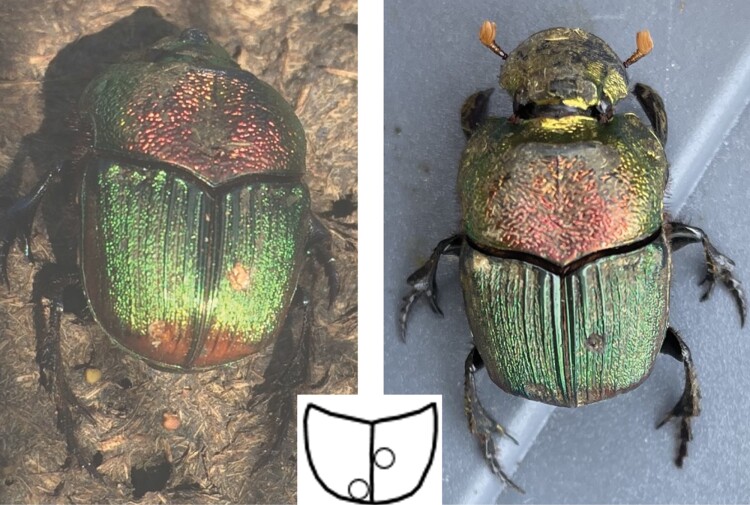
Recapture of a *Phanaeus vindex* after 338 days, 97 m away from the release site. Left panel: Capture #150 from June 8 2020 in trap 2 on Carpenter Farms recorded as a large female with a length of 20.29 mm and a width of 12.22 mm. Right panel: Capture #347 from October 7 2021 in trap 5 on Carpenter Farms recorded as a large female with a length of 20.20 mm and a width of 12.15 mm. The small insert shows the idealized tattoo pattern #59.

The minimum time interval between capture and recapture was 1 h (same day recaptures), and the maximum interval was 338 days. The distribution is extremely right skewed (30.6 ± 82.6, median: 7.0, Q1: 7.0, Q2: 14.0, Fig. 6). One large female was first captured and marked on 6 August 2020 and was recaptured on 10 July 2021. The identification was independently and double-blinded verified by Pedro da Silva and Sarah Rossi De Gasperis (personal communications) after being provided access to the uncommented photography database of all tattooed beetles (Fig. 7). This recapture indicates a multi-year lifespan and overwintering capacity of adult rainbow scarabs in the wild.

### Phenology

**Fig. 8.**
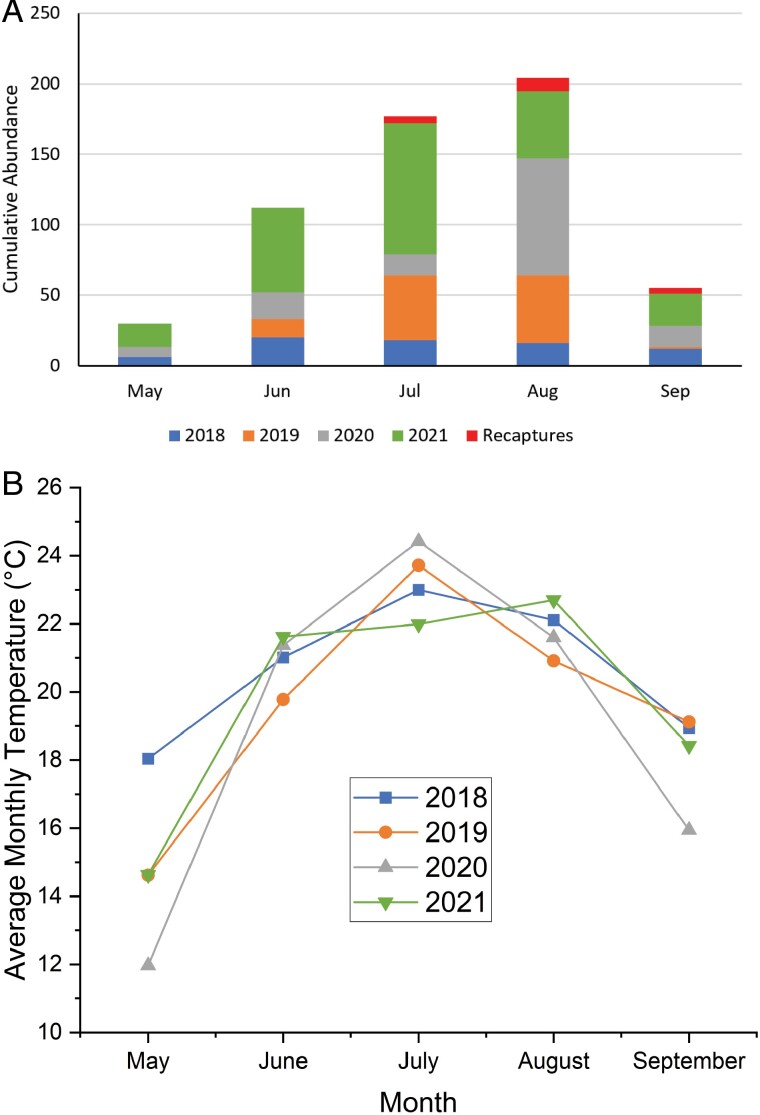
A) Phenology of rainbow scarabs in Adrian, MI over 4 consecutive years including the 3 yr of this study and the preceding year from previously published data (Wassmer 2020a, 2020b). In 2019, the study started on 6/9. B) Average monthly temperatures in the 3 yr of the current study and in the study by Wassmer (2020a, 2020b) using the same color coding.

During the 3 yr of the study, *P. vindex* occurred between mid-May and the end of September. However, the abundance of beetles over the months was slightly different between the years. In 2018, only a few beetles occurred in May, followed by even numbers of beetles in June, July, and August, and a slight reduction in September. In 2019, when collection did not start until June 9, most beetles occurred in July and August with only a third of the total number of beetles in June and only a single beetle in September. In 2020, almost double the number of beetles than in the combined other months occurred in August - while in 2021, most beetles occurred in July. In all years, recaptures only occurred between July and September and peaked in August (Fig. 8A). Beetle abundance was not significantly different by year (*F*_(3,18)_ = 2.37, *P* = 0.126) or month (*F*_(4,18)_ = 2.67, *P* = 0.089) and was not significantly correlated with average monthly temperatures (*r* = 0.429, *P* = 0.067) that are typically above 20°C between June and August (Fig. 8B).

### Population Structure

#### Sex ratio.

**Fig. 9.**
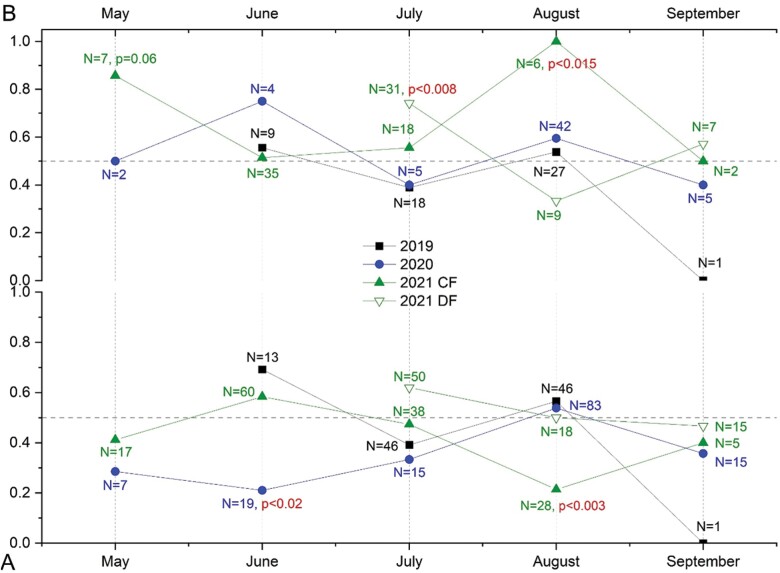
Lower panel A) Fluctuations of the adult sex ratio (*N* Male/*N* [male + female]) and upper panel B) fluctuations of the male minor/major ratio (N minor/N (minor + major)) over the sample months in all 3 sample years— and in 2021 separated for both farms. Sample sizes (*N*) are indicated for each sampling month. The dashed horizontal lines in (A) and (B) mark balanced ratios of 0.5. For most months, the sex ratio (B) was not statistically different from a balanced ratio but was female-biased in June 2020 and in August 2021 for Carpenter Farms (indicated by *P*-values in red). In 2019 and 2020, the sex ratio was not statistically different from a balanced ratio but was minor-biased on both farms in most months in 2021 reaching significance in July on Deline Farm and in August on Carpenter Farms (significant *P*-values indicated in red).

The sex ratio was 0.5 in 2019, 0.436 in 2020, and 0.498 in 2021 and was not significantly biased in any sample year (Chi-square tests, *P* > 0.05). However, in all years, the sex ratio fluctuated over every month with no apparent consistency between sample years besides a non-significant female bias in September (Fig. 9A). Significant female-bias occurred in June of 2020 (χ^2^ = 6.368, *P* = 0.016) and August of 2021 (χ^2^ = 5.565, *P* = 0.018).

#### Male major–minor dimorphism.

The male minor to major ratio was unbiased in 2019 (0.491) and in 2020 (0.569) (Fig. 9B). In 2021, the ratio showed a nonsignificant bias towards the minor morph (0.638, χ^*2*^ = 3.60, *P* = 0.058) and a nonsignificant female bias on DF (0.603, χ^2^ = 2.88, *P* = 0.090). Due to having double the number of males in 2021 compared to both 2020 and 2019, this also led to a significantly minor-biased morph ratio in the pooled years (0.575, χ^2^ = 5.115, *P* = 0.024). In 2019 and 2020, the morph ratio fluctuated between major- and minor-bias in all sample months whereas in 2021 on both farms most months showed a ratio towards the minor morph (Fig. 5B) and reached significance in July on DF (0.742, χ^*2*^ = 7.258, *P* < 0.008) and in August on CF (1.000, χ^2^ = 6.000, *P* < 0.015) where there was a nonsignificant minor-bias in May (0.857, χ^2^ = 3.572, *P* < 0.06).

#### Combined sex and male dimorphism ratios.

In 2020, the sex ratio showed the greatest female bias in June when minor males were more abundant than major males (Fig. 9). However, only the female bias was significant. In 2021 on CF, minor males were more abundant than major males when the sex ratio was female biased in May and August. However, in May only the bias towards minor males was significant but not the female bias. In August both biases were significant. In July 2021 on DF, minor males were more frequent than major males (*P* < 0.008) at a time when there was a nonsignificant male to female bias. In contrast to this, minor males were more abundant in September 2021 on DF, when the sex ratio bias was female biased. However, both biases were nonsignificant.

#### Population estimates.

**Fig. 10.**
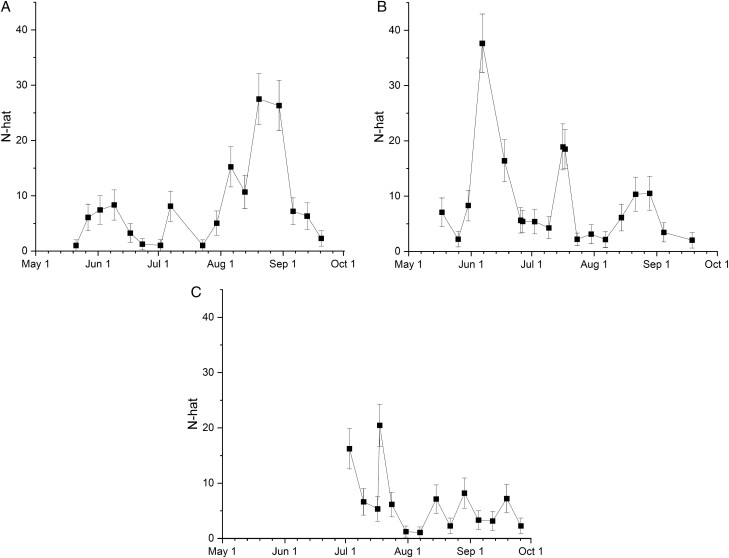
The daily population size estimate (N-hat) for all farms and years from the model *φ*(.)*p*(.)*pent*(t) with the apparent survival probability *φ* and the recapture probability *p* constant in time (.) and the time dependent probability of entering the population *pent* (t). Error bars show the 95% confidence intervals.

Daily population size estimates (N-hat) varied substantially between years and farm site (Fig. 10). The gross population estimate for Carpenter farms in 2020 was 458 ± 56 (94 ± 11 per ha) and in 2021 491 ± 57 rainbow scarabs (100 ± 12 per ha) while the gross population for the Deline farm was estimated at 217 ± 29 rainbow scarabs (13 per ha).

#### Dispersal.

**Fig. 11.**
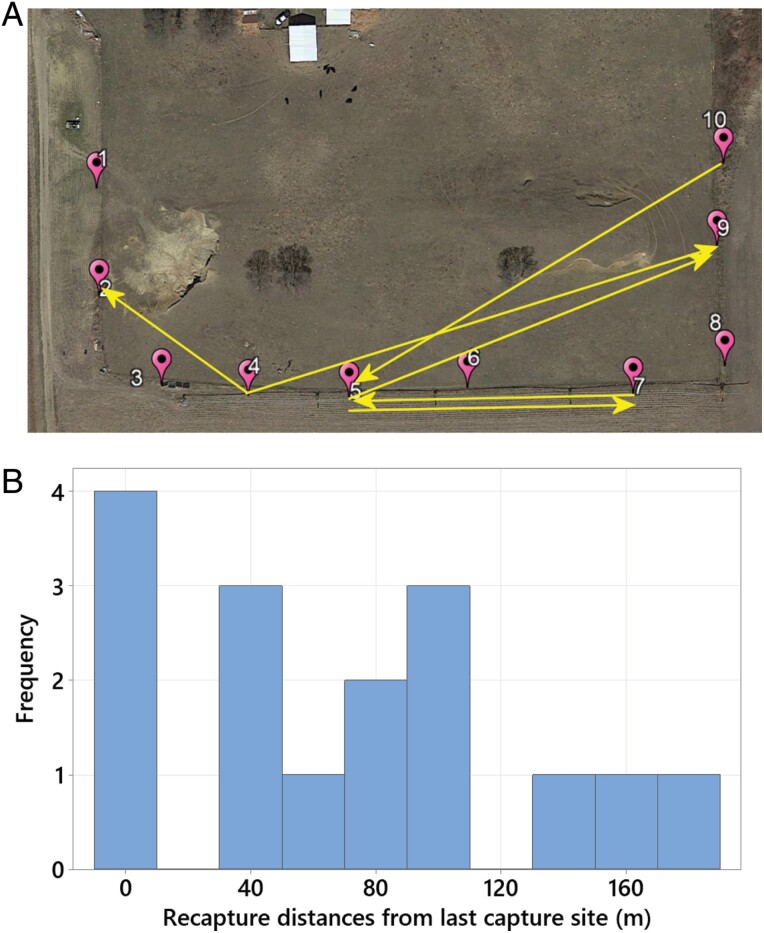
A) Dispersal events from Carpenter Farm in 2020. B) Histogram of all recapture distances relative to the last release site. Bin width is 20 m, labels are centered on bins.

Beetles were recaptured between 0 (recaptured in the same trap) and 178 m away from their original capture/release or previous recapture/release site. The mean distance between capture and recapture sites was 64.6 ± 57.6 m – the distribution showed a right skew (Fig. 11B). There were no sex difference in the distances of dispersals (females: 67.7 ± 58.8 vs. males 72.6 ± 61.2, *F*_(1, 15)_ = 0.03, *P* = 0.875). No marked beetles were recaptured on the closest neighboring pasture that was about 2.4 km away. A minor male dispersed at 103 meters in about 10 min, whereas a major male dispersed 178 m in 7 days. Another minor male was twice recaptured in the same trap after 7 days and again after 14 days.

## Discussion

### Morphometrics

Like 84% of insect species (Teder 2014), *Phanaeus vindex* is characterized by a female-biased sexual size dimorphism in which females are on average slightly but significantly larger and wider than males (Table 1, Fig. 1A). As in *Copris lunaris* (Kerman et al. 2018), minor and major morphs were clearly distinguished by horn length, pronotal width, and length with only minor overlaps (Fig. 1B). The allometry between width (or length) and horn length is between a smoothly curvilinear relationship reported for *Euoniticellus intermedius* (Knell 2009), and a sigmoidal relationship reported for *Onthophagus taurus* (Knell 2009) and *Copris lunaris* (Kerman et al. 2018) (Appendix S1: Supplementary Fig. S1).

### Recapture Rates

Our recapture rates of 1.9–3.8% (Table 2) fall within the lower end of all other MRR/CMR studies of dung beetles (1.5–41%) (Peck and Forsyth 1982, Roslin 2000, Cultid-Medina et al. 2015, da Silva and Hernández 2015, Villada-Bedoya and Cultid-Medina 2017, Barretto et al. 2019). As we used an almost identical trap design and marking technique, our lower recapture rates might be related to climatic and landscape differences. With the exception of the study of Roslin (2000), every comparable research (cited above) was conducted in tropical climates that were either lacking seasons or were only alternating between rainy and dry seasons and were situated in less fragmented landscapes with a higher percentage of forested areas. It is possible that both factors facilitated higher recapture rates. The only climatically similar study by Roslin (2000) was conducted in SW-Finland in a slightly cooler and more maritime climate than the current study and recorded the highest recapture rates of up to 41%. However, the study included only endocoprid (Doube 1990) and mainly dung dwelling Aphodiinae species (Sladecek et al. 2017), and was situated in a less fragmented – mainly pasture dominated landscape. These factors and the almost ideal sample design of 3 rectangular “simulated” pastures of 45 × 20 m separated from each other by only 300 m might explain the author’s much higher upper recapture rates that exceeded the upper ranges of all other dung beetle studies by at least 10%. Besides differences in climate, landscape, and functional ecology, another major factor that could have contributed to our relatively low capture rates is that our sampling interval of 1 wk was much longer than in the other studies that resampled after only 24 or 48 h or in a geometric progression after 24, 48, 96 h and so on. It is possible that many marked beetles did not stay on the pasture for a week after being marked and despite our pastures’ isolation in a fragmented landscape migrated away. We also ran relatively few traps that were not spread throughout the landscape but were confined to fence lines around or between pastures. A final difference between our study and almost all other MRR/CMR studies on dung beetles is our much smaller sampling area of just 4.9 and 16.3 ha, whereas the Meso- and South American studies were conducted on much larger landscapes of 80–760 ha. If the sample area is factored in, our per ha rates are 10× higher than any Meso- and South American study.

### Longevity and Overwintering

Most recaptures occurred in the next sampling after being marked and released (1 wk later) (Fig. 6). One large female was recaptured after 338 days and survived winter in Michigan as an adult (Fig. 7). When originally captured and marked in August 2020, this beetle was not teneral (soft-bodied) but already hardened suggesting that she may have been already overwintering before. This is only the second verified multiyear survival of an adult Scarabaeinae beetle in the wild (Cultid-Medina and Martínez-Quintero 2019), and the first outside the tropics in a climate with pronounced seasonality and cold winters. The female recaptured showed little wear of the clypeus (head shield) that is intensively used as a shovel by paracoprid relocating dung beetles like the rainbow scarab to fragment and bury the excrement. This might indicate the capacity to survive multiple years. Although the rainbow scarab rarely reaches body lengths of more than 20 mm, this species is still capable of a multiple-year life cycle, which was postulated for much larger beetles (>30 mm in body size) by Halffter and Matthews (1966). This may be due to being not a tropical but temperate species that occurs in Michigan in a slightly continental sub climate, which might facilitate slower multi-year life cycles similar to those assumed for many central Asian species (Siyazov 1913, Balthasar 1963).

### Phenology

During the 3 yr of the study, *P. vindex* occurred in a unimodal distribution between mid-May and the end of September (Fig. 8A), which corresponds well to a previous phenological study conducted in 2018 on Carpenter Farm (Wassmer 2020a). Beetle abundance was not significantly influenced by year or month but was marginally correlated with average monthly temperatures (Fig. 8B). The significance of climatic factors, especially temperature on dung beetle abundance and species richness has been reported in many studies, e.g., Vessby (2001), Davis (2002), Domínguez et al. (2015), Lobo and Cuesta (2021), Daoudi et al. (2022), Gotcha et al. (2022) and seems to even surpass the importance of dung availability (Gebert et al. 2020). Recaptures occurred only between July and September and peaked in August (Fig. 8A) suggesting that individuals caught early in the year are not staying on their parental pasture. However, this might just appear this way due to the lower number of beetles caught and marked early in the year. Closer to the Eastern seaboard, the species seems to occur earlier in the year and have an extended phenology into late fall (Price 2004, Price et al. 2012, Simons et al. 2018). More south, *P. vindex* might appear in early spring but disappears earlier in late summer (Bertone et al. 2005, Hinson 2011, Conover et al. 2019). There are some indications for a second generation at the Eastern seaboard and in Florida but given the size and relatively long larval development (Fincher 1972), it is more likely to assume a single yearly generation with some multi-year survivors and the fluctuations are likely caused by immigration and emigration and stochastic weather events.

### Population Structure

#### Sex ratio.

The sex ratio was not significantly sex-biased in any sample year. However, every year, the sex ratio fluctuated between the months with no apparent uniformity between years (Fig. 9A). Biased sex ratios may arise from different birth rates for the sexes (House et al. 2011, Lachowsky and Reid 2014), non-synchronized maturation (Ansari et al. 2006), differences in swarming time (Švestka 2018), differences between multiple generations (Christensen and Dobson 1976), may occur due to sex-dependent attraction to traps (Dutrillaux et al. 2014), or result from one sex being larger or differently shaped than the other if this difference contributes to higher survival rates (Lachowsky and Reid 2014). There are no clear patterns for the sex ratios of dung beetle populations reported so far as some species showed either a female bias (Dalgleish et al. 2005, House et al. 2011, Puker et al. 2014, Barretto et al. 2019), a balanced sex ratio of 0.5 (Hanski 1980, Garcia-Gonzalez and Simmons 2011, House et al. 2011, Buzatto et al. 2012) or were male-biased (Yamada et al. 2007, Reynolds and Byrne 2013, Puker et al. 2014, Barretto et al. 2019). However, some tribes of Scarabaeinae such as the Deltochilini were consistently described as female-biased (Puker et al. 2014, Barretto et al. 2019), while others such as the Coprini and Phanaeini were labelled as male-biased (Puker et al. 2014, Barretto et al. 2019). In burrowing beetles (paracoprids) such as Phanaeini, Coprini, and Onthophagini, males were reported to contribute little to parental care leaving the female responsible for most of the gallery excavation and construction and the provisioning of the nesting cells. This would allow males to allocate more time to compete with other males for access to females leading to a male-biased sex ratio (Halffter and Lopez 1977, Halffter and Edmonds 1982, Price and May 2009). Another suggested determinant for biased sex ratios is the possession or absence of horns. In a community of 14 African dung beetles, Pomfret and Knell (2008) found that hornless species tend to show female-biased sex ratios while horned species show balanced or male-biased ratio.

The rainbow scarab *P. vindex* is a horned Phanaeini and following the above predictions should have a male bias. In contrast to this, the overall sex ratios in every sample year were balanced and even reached a significant female-bias of almost 80% females in June 2020 and August 2021 (Fig. 5A). One reason for this discrepancy might be that unlike most other Phanaeini, female rainbow scarabs tend to be slightly but significantly larger than males (Table 1, Fig. 5A), which could favor a female biased sex ratio for this species (Lachowsky and Reid 2014). As the sexual size dimorphism is small, an overall balanced sex ratio might be favored unless it is influenced by other factors such as a biased male major-minor dimorphism.

#### Male major–minor dimorphism.

The male minor to major ratio was balanced in 2019 and in 2020 and the morph ratio fluctuated between major- and minor-biased months. In contrast to this, the male morph ratio fluctuated less in 2021 and most months were minor biased. The predominance of minor males in the current study corresponds well to the dominance of minor males in *O. taurus*, which typically exceed major males by a ratio of 4 to 1 (Hunt et al. 1999). In this species, the reproductive success of major males is about 4.5 times higher than that for minor males, which corresponds to the numerical dominance of the minor morph (Hunt and Simmons 2001). In *Onthophagus binodis*, 31% of males were minor (Simmons et al. 1999). In *P. vindex*, the minor morph also reaches ratios of 4:1 or higher (minor to all morphs ratio of 0.8, Fig. 5B) suggesting a reproductive dominance of the major morph at similar values. To my knowledge, there are no other studies reporting male morph ratios and none reported seasonal fluctuations of the ratio besides a remark by Eberhard (1982) that minor males of the dimorphic horned dynastinae beetle *Podischnus agenor* were more abundant than majors earlier in the season when females were still uncommon and that minors virtually disappeared later in the year. As this evidence combines the seasonality of both sex bias with male morph bias, we will discuss possible correlations of both factors in the next section.

#### Combined sex and male dimorphism ratios.

As predicted by Eberhard (1982), minor males of *Copris acutidens* were more abundant than major males early in the year and coincided partially with female bias (Akamine 2019). In contrast to this, dominance of minor *P. vindex* males occurred in all months and did only occasionally coincide with female dominance (Fig. 9AB). In *O. taurus*, House et al. (2011) found the same phenological coincidence of female and minor bias early in the year (Eberhard 1982, Akamine 2019), and postulated that females produce an excess of daughters when mating to minor males while producing a balanced sex ratio with major males. *O. taurus* is a species with a relatively fast ontogeny and phenological plasticity allowing it to sustain at least 2 generations per year in temperate climate regions (Wassmer 1994, 2020a). In contrast to this, *P. vindex* is clearly univoltine [Fig. 8A, Wassmer (2020a)] and is capable to overwinter as an adult beetle (Fig. 7). This may lead to less seasonally restricted pathways for minor males. The difference between *P. vindex* and *C. acutidens*, however, cannot be explained by this argument as both species are univoltine and most likely overwinter as adults (Bang et al. 2004, Akamine 2019). Nevertheless, even without the same phenological/voltinism pattern, it is possible that the fluctuations of the male morph ratio allow minor males to avoid conflict with major males (Eberhard 1982).

#### Population estimates.

Daily population size estimates (N-hat) varied substantially between years and farm site (Fig. 10) and gross population estimates were extremely low for both farms. Given the low recapture rate in this study, the only model we considered *a priori* was *φ*(.) *p*(.) *pent*(t), because we could not estimate a time variation in survival or catchability whereas we could for the recruitment rate as we captured many new individuals in each sampling event (Nicolas Schtickzelle, personal communication). We are aware that survival and catchability may not be constant through time which may lead to biased population size estimates through time. However, since these are the first population estimates for the rainbow scarab *P. vindex* or for any temperate dung beetle species in the world at all, we believe that our data might serve as an approximation and inspire future studies.

From working on Carpenter Farm for 4 yr, we believe that a head count of 450–500 beetles for this 4.9 ha farm (94–100 beetles per ha) that has no cattle present between May and October might be an overestimate. In contrary, the population estimate of the larger Deline Farm of 217 ± 29 rainbow scarabs (13 per ha) seems to be too low, especially since this farm is grazed by cattle all year around. From May to October of every year, the farmer of Carpenter Farm moved most/all cows to a more productive pasture. Only goats, ponies, and donkeys were present, ranging from 0 to 22 animals per 4.9 ha. On Deline Farm, 45–50 cattle are paddocked over 16.3 ha. These numbers result in 4.5 animals per ha on Carpenter Farm and 3.1 per ha on Deline Farm. However, if the rotating confinement of cattle into much smaller paddocks on Deline Farm is considered, the animal density causing a high dung pad density could increase substantially on an active paddock leading to an underestimate of the Deline Farm population, especially as the estimate was based on a single year between the months of July and October.

Due to the scarcity of studies into the population structure of dung beetles, we can only compare these numbers to studies from tropical Meso- and South America. Barretto et al. (2019) sampled a landscape in the mountains of tropical Mexico. Both sampled species, *Deltochilum mexicanum* and *Dichotomius satanas* showed daily population estimates of more than 4,000 beetles, which seems to be much higher than the 200–500 beetles’ population sizes in the current study. However, the authors sampled a much larger landscape of 126 ha (as to 4.9 and 16.3 ha) and used a much shorter trapping interval of just 24 h compared to 7 days, which both could have increased their recapture rate and therefore their population estimate. In addition, the researcher calculated their estimate using a different software (FSA: Simple Fisheries Stock Assessment Methods, Ogle et al. 2022), which is based on the traditional Cormack-Jolly-Seber model (Pollock et al. 1990) – and produced population estimates that are not directly comparable to our estimates based on the POPAN formulation in MARK. Nevertheless, if we relate the estimates of Barretto et al. (2019) to their sampling area, their population densities of 3.18 per ha are smaller than the 13 per ha for Deline Farm and 94–100 per ha on Carpenter Farm. Arellano et al. (2008) provide a monthly average population estimate for *Canthon cyanellus cyanellus* in S-Mexico of 5,322 ± 3,090 individuals based on the Jolly-Seber equations provided in on 263 ha and a sampling interval of 48 h, leading to a population density of 20 beetles per ha. Cultid-Medina et al. (2015) reported even higher maximum daily estimates of up to 30,000 *Dichotomius cf. alyattes* and *Oxysternon conspicillatum* from the foothills of tropical Columbia estimated by the FSA package (Ogle et al. 2022). The higher population estimate might have been again facilitated by a more frequent sampling interval of 24, 72, and 120 h in every month and their much larger sample area of 760 ha, bringing their population density down to 40 per ha. Their peak estimates correlated with the time right after the rainy season. In our area we did not find any substantial rainfall before the peak daily estimates for *P. vindex* (Fig. 10). A drastic example of the impact of the method on which population estimates are based upon can be seen by comparing all the above estimates to the oldest study providing dung beetle population estimates from tropical Ecuador (Peck and Forsyth 1982). The authors used the simple Lincoln index (Lincoln 1930), which is more appropriate for closed populations and received a population estimate of 169217 individuals, which when related to the relatively small size of their sampling area of just 89 ha, results in a population density of over 2000 dung beetles per ha.

#### Dispersal.

Most rainbow scarabs were recaptured in proximity of their release site with a maximum dispersal of only 178 m (Fig. 11A and B). No beetles dispersed between farms (distance between CF and DF is approx. 2.4 km). Male and female rainbow scarabs dispersed approximately equal distances and most recaptures occurred after 1–3 wk (Fig. 6). Although our sampling areas were much smaller, our sampling intervals were longer and the 2 farms were isolated and distant from each other, there are similarities between our dispersal results and the available reference studies cited above. In the only other study from a temperate climate, Roslin (2000) simulated pastures as rectangular arrays of dung pats measuring 45 × 20 m, separated from each other by narrow grassland strips of 300 m. In his study, 73% of all recaptured individuals were found within the simulated pasture on which they were released. Only 1% of all recaptured individuals were found in isolated dung pats placed approximately 1 km from these experimental pastures. Furthermore, the author found more movements between pastures in larger species, species with a specific preference for dung of a certain age, and in species that are cow dung and open pasture specialists. *P. vindex* is a relatively large species (approx. 18 mm long), clearly prefers early succession dung (Wassmer 2020b), is not specifically attracted to cow dung and a generalist occurring in both open and forested areas (Price et al. 2012, Simons et al. 2018). The combination of these characteristics should make *P. vindex* a good disperser.

As with the population estimates, the majority of reference studies were conducted in tropical Meso- and South America. Arellano et al. (2008) found a large fraction of recaptured *C. cyanellus* on the site of release. Males were found to move faster, possibly because they actively search for mates and the limited time that males stay within nests. In our study, we also saw a trend to faster movement by males, but our recapture numbers were too low and sampling intervals too long to be able to quantify this. In their study from tropical Colombia, Cultid-Medina et al. (2015) found that *O. conspicillatum* moved between 479 and 1,700 m within 24 h, while *D. cf. alyattes* moved 456–717 m within 24 h. Also from the Columbian Andes, Escobar and Chacón de Ulloa (2000) reported a maximum displacement distance of approximately 2 km by an individual of *Sulcophanaeus velutinus* after 1 month of being released, while an individual from *Dichotomius cf. quinquedens* was recaptured 7 months later at the site of the first capture. Finally, da Silva and Hernández (2015) found the furthest movements among dung beetle species in tropical coastal Brazil to be approx. 850 m with no sex bias. The estimated movement distance traveled by dung beetles in 48 h was 90 m and in 96 h was 93 m. The authors speculate that low recapture rates—like the rainbow scarab in our study—may not always be related to a low abundance of a species but could be caused by high dispersal rates, whereby species fly longer distances possibly due to a random distribution of ephemeral food resources. *P. vindex* seems to be a food generalist and was captured in pastures and forests (Price et al. 2012, Simons et al. 2018). It is possible that the current landscape mosaic provides scattered habitats and food sources in-between the highly fragmented pastures (Figs. 1B, Appendix S1: Supplementary Fig. S2). However, these habitats and food sources are most likely scarce, ephemeral, and unreliable.

Another reason for low recapture rates and low dispersal rates mentioned by the authors is that some paracoprid dung beetle species remain buried for long time periods while they are rearing offspring. What determined dispersal rates the most were the interactions between body size, diel or nocturnal activity, and dung relocation behavior (paracoprid/tunnelers vs. telocoprid/rollers) with large, diurnal tunnelers showing a greater mean movement rate than other species. *P. vindex* is a large, diurnal tunneler and should therefore be a good disperser.

### Relevance for Conservation Biology

The current study provides the first data on the sex ratio, male morph ratio, population size, dispersal, and longevity for the rainbow scarab, *P. vindex*. If our population size estimates of 200–500 beetles per pasture are correct, the local populations of the rainbow scarab are most likely not viable. Some specialists believe that any population of any species needs to amount to at least 5,000 individuals to be viable (Traill et al. 2010). However this “magic number” is disputed by others (Flather et al. 2011). As common agricultural practices continue to intensify and concentrate on cash crops, in the region especially corn and soybeans (Blayney 2004, Clay et al. 2020), cattle pastures become increasingly rare and highly fragmented in the landscape (Roslin and Koivunen 2001). Within a radius of 5 km around Carpenter Farm, there are only 5 other pastures with Deline Farm being the closest at 2.4 km (Appendix S1: Supplementary Fig. S2). Small populations with little connectivity between each other are known to be one of the most common reasons for the extirpation of dung beetle populations (Larsen et al. 2008, Numa et al. 2009). In addition, small scale cattle herders often move their cattle to more productive pastures from spring to late fall (Wassmer 2020b). This can leave pastures that provided habitat and food for dung beetles throughout the year for decades without a stable food source when they are most needed to provision their offspring (Wassmer 2020a). Another danger especially for dung beetle populations is the regular use of livestock dewormers (Verdú et al. 2015, Manning et al. 2018, Finch et al. 2020, Ambrožová et al. 2021, Konopka et al. 2022, González-Gómez et al. 2023). The combination of the stressors habitat loss, fragmentation, and unreliable food sources might also lead to more long distance dispersal. Arellano et al. (2008) speculated that dung beetles spend long periods of time below the ground allocating most energy into reproduction and only disperse if they need to. Living in highly fragmented habitats and/or being subjected to insufficient or unreliable food sources, on the other hand, may lead to higher dispersal rates that are more difficult to detect especially with a low number of traps, exclusively placed within pastures and long sampling intervals. As long-distance dispersals are energetically costly, they may lead to an increased mortality rate and lower reproduction if suitable habitats are rare and fragmented thus intensifying the danger of extirpation of the local populations of *P. vindex*.

What makes this case study on the populations of the iconic rainbow scarab in SE Michigan even more relevant is that the same land use practices that threaten the populations of this species are threatening the populations of many other species and reduce biodiversity and the efficiency of ecosystem services in many rural areas of the United States and world-wide. Integrating farm animals on crop fields or on adjacent paddocked pastures (Integrated Crop-Livestock Systems, land-sharing) within a small-patch diverse landscape mosaic increases soil fertility (Rakkar and Blanco-Canqui 2018, Sartor et al. 2019, Galindo et al. 2020) and may reduce greenhouse gas emissions relative to concentrated beef and dairy operation (Ghahramani and Bowran 2018, Yang et al. 2022). Small cattle herders contributing to such systems should be supported as land stewards and receive supplementary funding similar to the USDA Conservation Reserve Program (CRP) for growing prairie strips (Riffell et al. 2008, McMinn-Sauder et al. 2020, USDA Farm Service Agency 2020).

Future studies should include all pastures within an area, implement traps on transects between pastures including forested areas and suburban settlements, sample in shorter intervals, e.g., each month after 24, 72, and 120 h. It would also be interesting to track the dispersal of individual tagged beetles, e.g., by drone-assisted harmonic radar tracking (Lavrenko et al. 2021, Woodgate et al. 2021) and relocate beetles from one pasture to another to see if there are any homing tendencies.

## Supplementary Material

iead050_suppl_Supplementary_Material

iead050_suppl_Supplementary_Video_S1

## Data Availability

Data from this study are available from the Figshare Repository (Wassmer 2023).

## References

[CIT0001] Akamine M. Size-dependent seasonal activity for males of the dung beetle *Copris acutidens* (Coleoptera: Scarabaeidae). Can Entomol. 2019:151(6):757–767. 10.4039/tce.2019.55

[CIT0002] Ambrožová L , Sládeček FXJ, Zítek T, Perlík M, Kozel P, Jirků M, Čížek L. Lasting decrease in functionality and richness: effects of ivermectin use on dung beetle communities. Agric Ecosyst Environ. 2021:321:107634. 10.1016/j.agee.2021.107634

[CIT0003] Ancona S , Dénes FV, Krüger O, Székely T, Beissinger SR. Estimating adult sex ratios in nature. Philos Trans R Soc B Biol Sci. 2017:372:20160313. 10.1098/rstb.2016.0313 PMC5540855 28760756

[CIT0004] Ansari MA , Casteels H, Tirry L, Moens M. Biology of *Hoplia philanthus* (Col., Scarabaeidae, Melolonthinae): a new and severe pest in Belgian Turf. Environ Entomol. 2006:35(6):1500–1507. 10.1093/ee/35.6.1500

[CIT0005] Arellano L , León-Cortés JL, Ovaskainen O. Patterns of abundance and movement in relation to landscape structure: a study of a common scarab (*Canthon cyanellus cyanellus*) in Southern Mexico. Landsc Ecol. 2008:23:69–78.

[CIT0006] Balthasar V. Monographie der Scarabaeidae und Aphodiidae der palaearktischen und orientalischen Region: Coleoptera: Lamellicornia. Prague (Czech Republic): Tschechoslowakische Akademie der Wissenschaften; 1963.

[CIT0007] Bang HS , Kwon OS, Hwang SJ, Mah YI, Watdhaugh KG. Developmental biology and phenology of a Korean native dung beetle, *Copris ochus* (Motschulsky) (Coleoptera: Scarabaeidae). Coleopt Bull. 2004:58(4):522–533. 10.1649/662

[CIT0008] Barretto JW , Cultid-Medina CA, Escobar F. Annual abundance and population structure of two dung beetle species in a human-modified landscape. Insects. 2019:10:2. 10.3390/insects10010002 PMC6358878 30597891

[CIT0009] Bertone M , Green J, Washburn S, Poore M, Sorenson C, Watson DW. Seasonal activity and species composition of dung beetles (Coleoptera: Scarabaeidae and Geotrupidae) inhabiting cattle pastures in North Carolina. Ann Entomol Soc Am. 2005:98(3):309–321. 10.1603/0013-8746(2005)098[0309:saasco]2.0.co;2.\

[CIT0010] Blayney D. 2004. The changing landscape of U.S. milk production. Statistical Bulletin No. SB-978. Washington (DC): United States Department of Agriculture, Economic Research Service.

[CIT0011] Buzatto BA , Tomkins JL, Simmons LW. Maternal effects on male weaponry: female dung beetles produce major sons with longer horns when they perceive higher population density. BMC Evol Biol. 2012:12:118. 10.1186/1471-2148-12-118 22823456 PMC3506554

[CIT0012] Cardoso P , Barton PS, Birkhofer K, Chichorro F, Deacon C, Fartmann T, Fukushima CS, Gaigher R, Habel JC, Hallmann CA, et al. Scientists’ warning to humanity on insect extinctions. Biol Conserv. 2020:242:108426. 10.1016/j.biocon.2020.108426

[CIT0013] Christensen CM , Dobson RC. Biological and ecological studies on *Aphodius distinctus* (Mueller) (Coleoptera: Scarabaeidae). Am Midl Nat. 1976:95(1):242–249. 10.2307/2424257

[CIT0014] Clay N , Garnett T, Lorimer J. Dairy intensification: drivers, impacts and alternatives. Ambio. 2020:49(1):35–48. 10.1007/s13280-019-01177-y 31055793 PMC6888798

[CIT0015] Conover D , Dubeux J, Martini X. Phenology, distribution, and diversity of dung beetles (Coleoptera: Scarabaeidae) in North Florida’s pastures and forests. Environ Entomol. 2019:48(4):847–855. 10.1093/ee/nvz068 31188428

[CIT0016] Cultid-Medina CA , Martínez-Quintero B. More than 5 years! An unusually long-lived dung beetle (Scarabaeinae) in an Andean agricultural landscape. Neotrop Entomol. 2019:48(3):522–526. 10.1007/s13744-019-00673-w 30779048

[CIT0017] Cultid-Medina CA , Martínez-Quintero BG, Escobar F, de Ulloa PC. Movement and population size of two dung beetle species in an Andean agricultural landscape dominated by sun-grown coffee. J Insect Conserv. 2015:19(4):617–626. 10.1007/s10841-015-9784-3

[CIT0018] Dalgleish EA , Elgar MA, Dalgleish EA, Elgar MA. Breeding ecology of the rainforest dung beetle *Cephalodesmius armiger* (Scarabaeidae) in Tooloom National Park. Aust J Zool. 2005:53:95–102.

[CIT0019] Daoudi L , Chavanon G, Taybi AF, Mabrouki Y. Composition and phenology of the beetle community (Coleoptera: Scarabaeoidea, Staphylinidae, Histeridae, Hydrophilidae) associated to dung of equines in an arid environment. Ann Soc Entomol Fr NS. 2022:58:155–164.

[CIT0020] Davis A. L. V. Dung beetle diversity in South Africa: influential factors, conservation status, data inadequacies and survey design. Afr Entomol. 2002:10:53–65.

[CIT0021] deCastro-Arrazola I , Andrew NR, Berg MP, Curtsdotter A, Lumaret J-P, Menéndez R, Moretti M, Nervo B, Nichols ES, Sánchez-Piñero F, et al. A trait-based framework for dung beetle functional ecology. J Anim Ecol. 2023:92(1):44–65. 10.1111/1365-2656.13829. 36443916 PMC10099951

[CIT0022] Dickey AM. 2007. Population genetics of *Phanaeus vindex* and *P. difformis* and congruence with morphology across a geographic zone of species overlap. Arlington, TX: University of Texas at Arlington. [accessed 2022 Aug 8]. https://rc.library.uta.edu/uta-ir/handle/10106/381.

[CIT0023] Domínguez D , Marín-Armijos D, Ruiz C. Structure of dung beetle communities in an altitudinal gradient of neotropical dry forest. Neotrop Entomol. 2015:44(1):40–46. 10.1007/s13744-014-0261-6. 26013011

[CIT0024] Doube BM. A functional classification for analysis of the structure of dung beetle assemblages. Ecol Entomol. 1990:15(4):371–383. 10.1111/j.1365-2311.1990.tb00820.x.

[CIT0025] Dutrillaux B , Pluot-Sigwalt D, Dutrillaux A-M. Unbalanced sex ratio and triploidy in the genus *Cyclocephala* (Coleoptera: Scarabaeoidea: Dynastidae) in the Lesser Antilles: An example of parthenogenesis on islands? EJE. 2014:111:313–319.

[CIT0026] Eberhard WG. Beetle horn dimorphism: making the best of a bad lot. Am Nat. 1982:119(3):420–426. 10.1086/283920.

[CIT0027] Edmonds WD. Revision of Phanaeus Macleay, a New World genus of scarabaeine dung beetles (Coleoptera: Scarabaeidae, Scarabaeinae). Contrib Sci. 1994:443:1–105.

[CIT0028] Escobar FS , Chacón de Ulloa P. Distribución espacial y temporal en un gradiente de sucesión de la fauna de coleópteros coprófagos (Scarabaeinae, Aphodiinae) en un bosque tropical montano, Nariño – Colombia. Rev Biol Trop. 2000:48:961–975. 11487941

[CIT0029] Finch D , Schofield H, Floate KD, Kubasiewicz LM, Mathews F. Implications of endectocide residues on the survival of aphodiine dung beetles: a meta-analysis. Environ Toxicol Chem. 2020:39(4):863–872. 10.1002/etc.4671 32181912

[CIT0030] Flather CH , Hayward GD, Beissinger SR, Stephens PA. Minimum viable populations: is there a “magic number” for conservation practitioners?. Trends Ecol Evol. 2011:26(6):307–316. 10.1016/j.tree.2011.03.001 21458878

[CIT0121] Fincher GT . Notes on the biology of Phanaeus vindex (Coleoptera: Scarabaeidae). J Georgia Entomol Soc. 1972:7:128–133.

[CIT0031] Foster CW , Kelly C, Rainey JJ, Holloway GJ. Effects of urbanisation and landscape heterogeneity mediated by feeding guild and body size in a community of coprophilous beetles. Urban Ecosyst. 2020:23(5):1063–1077. 10.1007/s11252-020-00997-1

[CIT0033] Galindo FS , Delate K, Heins B, Phillips H, Smith A, Pagliari PH. Cropping system and rotational grazing effects on soil fertility and enzymatic activity in an integrated organic crop-livestock system. Agronomy. 2020:10(6):803. 10.3390/agronomy10060803

[CIT0034] Garcia-Gonzalez F , Simmons LW. Good genes and sexual selection in dung beetles (*Onthophagus taurus*): genetic variance in egg-to-adult and adult viability. PLoS One. 2011:6(1):e16233. 10.1371/journal.pone.0016233 21267411 PMC3022759

[CIT0035] Gebert F , Steffan-Dewenter I, Moretto P, Peters MK. Climate rather than dung resources predict dung beetle abundance and diversity along elevational and land use gradients on Mt. Kilimanjaro. J Biogeogr. 2020:47:371–381.

[CIT0036] Ghahramani A , Bowran D. Transformative and systemic climate change adaptations in mixed crop-livestock farming systems. Agric Syst. 2018:164:236–251.

[CIT0037] González-Gómez L , González-Tokman D, García JH, Lira-Noriega A, Escobar F. Influence of landscape and livestock management on dung beetle diversity in tropical cattle pastures. Biodivers Conserv. 2023:32(5):1687–1707. 10.1007/s10531-023-02571-5

[CIT0038] Gotcha N , Cuthbert RN, Machekano H, Nyamukondiwa C. Density-dependent ecosystem service delivery under shifting temperatures by dung beetles. Sci Total Environ. 2022:807(Pt 1):150575. 10.1016/j.scitotenv.2021.150575 34634717

[CIT0122] Halffter G , Edmonds WD. The nesting behavior of dung beetles (Scarabaeinae): an ecological and evolutive approach. Instituto de Ecologia, Mexico, D.F; 1982.

[CIT0039] Halffter G , Halffter V, I LG. *Phanaeus* behavior: food transportation and bisexual cooperation. Environ Entomol. 1974:3:341–345.

[CIT0040] Halffter G , Lopez YG. Development of the ovary and mating behavior in *Phanaeus*. Ann Entomol Soc Am. 1977:70:203–213.

[CIT0041] Halffter G , Matthews EG. 1966. The natural history of dung beetles of the subfamily Scarabaeinae (Coleoptera, Scarabaeidae). Mexico (DF): Sociedad Mexicana de Entomologia.

[CIT0042] Hanski I. Spatial patterns and movements in coprophagous beetles. Oikos. 1980:34(3):293–310. 10.2307/3544289

[CIT0043] Haynes RJ , Williams PH. Nutrient cycling and soil fertility in the grazed pasture ecosystem. Adv Agron. 1993:49:119–199.

[CIT0044] Henderson PA. Southwood’s ecological methods. 5th ed. New York (NY): Oxford University Press; 2021a.

[CIT0046] Hinson KR. Species diversity and seasonal abundance of Scarabaeoidea at four locations in South Carolina. Entomology. 2011.

[CIT0047] House CM , Simmons LW, Kotiaho JS, Tomkins JL, Hunt J. Sex ratio bias in the dung beetle *Onthophagus taurus*: adaptive allocation or sex-specific offspring mortality? Evol Ecol. 2011:25:363–372.

[CIT0048] Hunt J , Kotiaho JS, Tomkins JL. Dung pad residence time covaries with male morphology in the dung beetle *Onthophagus taurus*. Ecol Entomol. 1999:24(2):174–180. 10.1046/j.1365-2311.1999.00192.x

[CIT0049] Hunt J , Simmons LW. Status-dependent selection in the dimorphic beetle *Onthophagus taurus*. Proc R Soc Lond B Biol Sci. 2001:268:2409–2414. 10.1098/rspb.2001.1758 PMC1088894 11747558

[CIT0050] Kerman K , Roggero A, Rolando A, Palestrini C. Evidence for male horn dimorphism and related pronotal shape variation in *Copris lunaris* (Linnaeus, 1758) (Coleoptera: Scarabaeidae, Coprini). Insects. 2018:9(3):108. 10.3390/insects9030108 30135396 PMC6164466

[CIT0051] Kirkpatrick WH , Sheldon KS. Experimental increases in temperature mean and variance alter reproductive behaviours in the dung beetle *Phanaeus vindex*. Biol Lett. 2022:18(7):20220109. 10.1098/rsbl.2022.0109 35857889 PMC9256084

[CIT0052] Knell RJ. On the analysis of non-linear allometries. Ecol Entomol. 2009:34(1):1–11. 10.1111/j.1365-2311.2008.01022.x

[CIT0053] Konopka JK , Chatterjee P, LaMontagne C, Brown J. Environmental impacts of mass drug administration programs: exposures, risks, and mitigation of antimicrobial resistance. Infect Dis Poverty. 2022:11(1):78. 10.1186/s40249-022-01000-z 35773680 PMC9243877

[CIT0054] Krebs CJ. Ecological methodology. 2nd ed. Menlo Park (CA): Benjamin/Cummings; 1999.

[CIT0056] Lachowsky LE , Reid ML. Developmental mortality increases sex-ratio bias of a size-dimorphic bark beetle. Ecol Entomol. 2014:39(3):300–308. 10.1111/een.12108 25400320 PMC4207193

[CIT0057] Larsen TH , Lopera A, Forsyth A. Understanding trait-dependent community disassembly: dung beetles, density functions, and forest fragmentation. Conserv Biol. 2008:22(5):1288–1298. 10.1111/j.1523-1739.2008.00969.x 18616744

[CIT0058] Lavrenko A , Barry Z, Norman R, Frazer C, Ma Y, Woodward G, Pawson S. Autonomous swarm of UAVs for tracking of flying insects with harmonic radar. In: 2021 Ieee 93rd Veh. Technol. Conf. Vtc2021-Spring. New York (NY): IEEE; 2021.

[CIT0059] Lincoln FC. Calculating waterfowl abundance on the basis of banding returns. Washington (DC): US Department of Agriculture; 1930.

[CIT0060] Liu Z , He C, Wu J. The relationship between habitat loss and fragmentation during urbanization: an empirical evaluation from 16 world cities. PLoS One. 2016:11(4): e0154613. 10.1371/journal.pone.0154613 27124180 PMC4849762

[CIT0061] Lobo JM , Cuesta E. Seasonal variation in the diel activity of a dung beetle assemblage. PeerJ. 2021:9:e11786. 10.7717/peerj.11786 34306833 PMC8280883

[CIT0062] Maldonado MB , Aranibar JN, Serrano AM, Chacoff NP, Vázquez DP. Dung beetles and nutrient cycling in a dryland environment. CATENA. 2019:179:66–73. 10.1016/j.catena.2019.03.035

[CIT0063] Manning P , Lewis OT, Beynon SA. Effects of the veterinary anthelmintic moxidectin on dung beetle survival and dung removal. Entomol Exp Appl. 2018:166(10):810–817. 10.1111/eea.12730

[CIT0064] McMinn-Sauder H , Richardson R, Eaton T, Smith M, Johnson R. Flowers in conservation reserve program (CRP) pollinator plantings and the upper midwest agricultural landscape supporting honey bees. Insects. 2020:11(7):405. 10.3390/insects11070405 32629811 PMC7411617

[CIT0065] Nichols E , Spector S, Louzada J, Larsen T, Amezquita S, Favila ME. Ecological functions and ecosystem services provided by Scarabaeinae dung beetles. Biol Conserv. 2008:141:1461–1474.

[CIT0066] Numa C , Verdú JR, Sánchez A, Galante E. Effect of landscape structure on the spatial distribution of Mediterranean dung beetle diversity. Divers Distrib. 2009:15(3):489–501. 10.1111/j.1472-4642.2009.00559.x

[CIT0067] Ogle D , Doll J, Wheeler P, Dinno A. FSA: simple fisheries stock assessment methods. 2022. [accessed 2022 Jul 5]. https://CRAN.R-project.org/package=FSA.

[CIT0068] Paris T , Rohde B, Kaufman PE. Rainbow scarab Phaneaus vindex Macleay (Insecta: Coleoptera: Scarabaeidae). EENY567/IN1003, 7/2013. EDIS; 2013. [accessed 2023 Jun 20]. https://journals.flvc.org/edis/article/view/121106.

[CIT0069] Peck SB , Forsyth A. Composition, structure, and competitive behaviour in a guild of Ecuadorian rain forest dung beetles (Coleoptera; Scarabaeidae). Can J Zool. 1982:60(7):1624–1634. 10.1139/z82-213.

[CIT0070] Pokhrel MR , Cairns SC, Andrew NR. Dung beetle species introductions: when an ecosystem service provider transforms into an invasive species. PeerJ. 2020:8:e9872. 10.7717/peerj.9872. 33062417 PMC7531351

[CIT0071] Pollock KH , Nichols JD, Brownie C, Hines JE. Statistical inference for capture–recapture experiments. Wildl Monogr 107: 1990:3–97.

[CIT0072] Pomfret JC , Knell RJ. Crowding, sex ratio and horn evolution in a South African beetle community. Proc R Soc B Biol Sci. 2008:275:315–321. 10.1098/rspb.2007.1498 PMC2593729 18048281

[CIT0073] Price DL. Species diversity and seasonal abundance of scarabaeoid dung beetles (Coleoptera: Scarabaeidae, Geotrupidae and Trogidae) attracted to cow dung in central New Jersey. J N Y Entomol Soc. 2004:112(4):334–347. 10.1664/0028-7199(2004)112[0334:sdasao]2.0.co;2

[CIT0074] Price DL , Brenneman LM, Johnston RE. Dung beetle (Coleoptera: Scarabaeidae and Geotrupidae) Communities of Eastern Maryland. Proc Entomol Soc Wash. 2012:114(1):142–151. 10.4289/0013-8797.114.1.142

[CIT0075] Price DL , May ML. Behavioral ecology of Phanaeus dung beetles (Coleoptera: Scarabaeidae): review and new observations. Acta Zool Mex. 2009:25:211–238.

[CIT0076] Puker A , Correa CMA, Korasaki V. Deltochilini and Phanaeini dung beetles (Coleoptera: Scarabaeidae: Scarabaeinae) in introduced and native ecosystems of Brazil. J Nat Hist. 2014:48:2105–2116.

[CIT0077] Rakkar MK , Blanco-Canqui H. Grazing of crop residues: impacts on soils and crop production. Agric Ecosyst Environ. 2018:258:71–90.

[CIT0078] Rasmussen J. L. The influence of horn and body size on the reproductive behavior of the horned rainbow scarab beetle *Phanaeus difformis* (Coleoptera: Scarabaeidae). J Insect Behav. 1994:7(1):67–82. 10.1007/bf01989828

[CIT0079] Reynolds C , Byrne MJ. Alternate reproductive tactics in an African dung beetle, *Circellium bacchus* (Scarabeidae). J Insect Behav. 2013:26:440–452.

[CIT0080] Ridsdill-Smith TJ , Hayles L. Stages of bush fly, *Musca vetustissima* (Diptera: Muscidae), killed by scarabaeine dung beetles (Coleoptera: Scarabaeidae) in unfavourable cattle dung. Bull Entomol Res. 1990:80:473–478.

[CIT0081] Riffell S , Scognamillo D, Burger LW. Effects of the conservation reserve program on northern bobwhite and grassland birds. Environ Monit Assess. 2008:146(1–3):309–323. 10.1007/s10661-007-0082-8 18175201

[CIT0082] Roslin T. Dung beetle movements at two spatial scales. Oikos. 2000:91(2):323–335. 10.1034/j.1600-0706.2000.910213.x

[CIT0083] Roslin T , Koivunen A. Distribution and abundance of dung beetles in fragmented landscapes. Oecologia. 2001:127(1):69–77. 10.1007/s004420000565 28547171

[CIT0084] Sands B , Wall R. Dung beetles reduce livestock gastrointestinal parasite availability on pasture. J Appl Ecol. 2017:54:1180–1189.

[CIT0085] Sartor LR , Sandini IE, Carvalho PCF, Ruthes BES. Soil fertility in crop-livestock system subjected to nitrogen fertilization and grazing. J Agric Sci. 2019:11:p121.

[CIT0086] Schwarz CJ , Arnason AN. A General methodology for the analysis of capture-recapture experiments in open populations. Biometrics. 1996:52(3):860. 10.2307/2533048

[CIT0087] Seber GAF. Estimation of animal abundance. 2nd ed. Caldwell (NJ): The Blackburn Press; 2002.

[CIT0088] da Silva PG , Hernández MIM. Spatial patterns of movement of dung beetle species in a tropical forest suggest a new trap spacing for dung beetle biodiversity studies. PLoS One. 2015:10:e0126112. 25938506 10.1371/journal.pone.0126112 PMC4418735

[CIT0089] Simkin RD , Seto KC, McDonald RI, Jetz W. Biodiversity impacts and conservation implications of urban land expansion projected to 2050. Proc Natl Acad Sci USA. 2022:119(12):e2117297119. 10.1073/pnas.2117297119 35286193 PMC8944667

[CIT0090] Simmons LW , Tomkins JL, Hunt J. Sperm competition games played by dimorphic male beetles. Proc R Soc Lond B Biol Sci. 1999:266:145–150. 10.1098/rspb.2000.1177 PMC1690708 11007331

[CIT0091] Simons P , Molina M, Hagadorn MA, Price DL. Monitoring of dung beetle (Scarabaeidae and Geotrupidae) activity along Maryland’s coastal plain. Northeast Nat. 2018:25(1):87–100. 10.1656/045.025.0108

[CIT0092] Siyazov MM. K biologii zhukov navoznikov (Coleoptera, Scarabaeidae). Russ Rev OJ Entomol. 1913:13:113–131.

[CIT0093] Slade EM , Kirwan L, Bell T, Philipson CD, Lewis OT, Roslin T. The importance of species identity and interactions for multifunctionality depends on how ecosystem functions are valued. Ecology. 2017:98(10):2626–2639. 10.1002/ecy.1954 28722121

[CIT0094] Slade EM , Riutta T, Roslin T, Tuomisto HL. The role of dung beetles in reducing greenhouse gas emissions from cattle farming. Sci Rep. 2016a:6:18140. 10.1038/srep18140 26728164 PMC4700445

[CIT0095] Slade EM , Roslin T, Santalahti M, Bell T. Disentangling the “brown world” faecal–detritus interaction web: dung beetle effects on soil microbial properties. Oikos. 2016b:125:629–635.

[CIT0096] Sladecek FXJ , Hrcek J, Klimes P, Konvicka M. Interplay of succession and seasonality reflects resource utilization in an ephemeral habitat. Acta Oecol. 2013:46:17–24. 10.1016/j.actao.2012.10.012

[CIT0097] Sladecek FXJ , Segar ST, Lee C, Wall R, Konvicka M. Temporal segregation between dung-inhabiting beetle and fly species. PLoS One. 2017:12(1):e0170426. 10.1371/journal.pone.0170426 28107542 PMC5249136

[CIT0098] SoilWeb-Earth. SoilWeb Earth. 2022. https://doi.org/https://casoilresource.lawr.ucdavis.edu/soilweb-apps/.

[CIT0099] Stanbrook R , King JR. Dung beetle community composition affects dung turnover in subtropical US grasslands. Ecol Evol. 2022:12(2):e8660. 10.1002/ece3.8660 35228864 PMC8861836

[CIT0100] Švestka M. Distribution of tribes of cockchafers of the genus Melolontha in forests of the Czech Republic and the dependence of their swarming on temperature; 2018.

[CIT0101] Teder T. Sexual size dimorphism requires a corresponding sex difference in development time: a meta-analysis in insects. Funct Ecol. 2014:28:479–486.

[CIT0102] Tocco C , Foster J, Venter N, Cowie B, Marlin D, Byrne M. Elevated atmospheric CO2 adversely affects a dung beetle’s development: Another potential driver of decline in insect numbers?. Glob Change Biol. 2021:27(19):4592–4600. 10.1111/gcb.15804 34265139

[CIT0103] Traill LW , Brook BW, Frankham RR, Bradshaw CJA. Pragmatic population viability targets in a rapidly changing world. Biol Conserv. 2010:143:28–34.

[CIT0104] USDA Farm Service Agency. Conservation reserve program. Natl.-Content; 2020. https://doi.org/https://fsa.usda.gov/programs-and-services/conservation-programs/conservation-reserve-program/index

[CIT0105] USDA. Dairy 2014. dairy cattle management practices in the United States, 2014 (no. February 2016, report 1). Fort Collins (CO): United States Department of Agriculture, Animal and Plant Health Inspection Service, Veterinary Services, National Animal Health Monitoring System; 2016.

[CIT0106] Verdú JR , Cortez V, Ortiz AJ, González-Rodríguez E, Martinez-Pinna J, Lumaret J-P, Lobo JM, Numa C, Sánchez-Piñero F. Low doses of ivermectin cause sensory and locomotor disorders in dung beetles. Sci Rep. 2015:5:13912. 10.1038/srep13912 26350768 PMC4563563

[CIT0107] Verdú JR , Sánchez-Piñero F, Lobo JM, Cortez V. Evaluating long-term ivermectin use and the role of dung beetles in reducing short-term CH4 and CO2 emissions from livestock faeces: a mesocosm design under Mediterranean conditions. Ecol Entomol. 2020:45:109–120.

[CIT0108] Vessby K. Habitat and weather affect reproduction and size of the dung beetle *Aphodius fossor*. Ecol Entomol. 2001:26(4):430–435. 10.1046/j.1365-2311.2001.00331.x

[CIT0109] Villada-Bedoya S , Cultid-Medina CA. Estructura poblacional de dos especies de Dichotomius Hope, 1838 (Coleoptera: Scarabaeinae) en un paisaje cafetero de Los Andes Occidentales de Colombia, Risaralda. Bol Cient Cent Mus Mus Hist Nat. 2017:21:188–198.

[CIT0110] Wassmer T. Seasonality of coprophagous beetles in the Kaiserstuhl area near Freiburg (Sw Germany) including the winter months. Acta Oecol Int J Ecol. 1994:15:607–631.

[CIT0111] Wassmer T. Attractiveness of cattle dung to coprophilous beetles (Coleoptera: Scarabaeoidea and Sphaeridiinae) and their segregation during the initial stages of the heterotrophic succession on a pasture in southeast Michigan. J Insect Sci. 2020b:20:(3):1–15. 10.1093/jisesa/ieaa040 PMC7273520 32501502

[CIT0112] Wassmer T. Phenological patterns and seasonal segregation of coprophilous beetles (Coleoptera: Scarabaeoidea and Hydrophilidae) on a cattle farm in SE-Michigan, United States throughout the year. Front Ecol Evol. 2020a:8:293.

[CIT0112a] Wassmer T , Armstrong E. Capture-mark-release (mark-release-recapture) records of rainbow scarabs (phanaeus vindex) from two farms in SE-Michigan. Figshare. Dataset; 2023. 10.6084/m9.figshare.22732256.v2

[CIT0113] Watling JI , Arroyo-Rodríguez V, Pfeifer M, Baeten L, Banks-Leite C, Cisneros LM, Fang R, Hamel-Leigue AC, Lachat T, Leal IR, et al. Support for the habitat amount hypothesis from a global synthesis of species density studies. Ecol Lett. 2020:23(4):674–681. 10.1111/ele.13471. 32043741

[CIT0114] Weatherbase. Weatherbase. 2022. https://doi.org/www.weatherbase.com.

[CIT0115] White GC , Burnham KP. Program MARK: survival estimation from populations of marked animals. Bird Study. 1999:46(supp1):S120–S139. 10.1080/00063659909477239

[CIT0116] White GC , Cooch EG. Program MARK: a gentle introduction. 19th ed. 2019.

[CIT0117] Wilson K , Hardy ICW. Statistical analysis of sex ratios: an introduction. In: Hardy ICW, editor. Sex ratios concepts res. methods. Cambridge: Cambridge University Press; 2002. p. 48–92.

[CIT0118] Woodgate JL , Makinson JC, Rossi N, Lim KS, Reynolds AM, Rawlings CJ, Chittka L. Harmonic radar tracking reveals that honeybee drones navigate between multiple aerial leks. iScience. 2021:24(6):102499. 10.1016/j.isci.2021.102499 34308279 PMC8257961

[CIT0119] Yamada D , Imura O, Shi K, Shibuya T. Effect of tunneler dung beetles on cattle dung decomposition, soil nutrients and herbage growth. Grassl Sci. 2007:53(2):121–129. 10.1111/j.1744-697x.2007.00082.x

[CIT0120] Yang G , Li J, Liu Z, Zhang Y, Xu X, Zhang H, Xu Y. Research trends in crop-livestock systems: a bibliometric review. Int J Environ Res Public Health. 2022:19(14):8563. 10.3390/ijerph19148563 35886413 PMC9318012

