# Supplementary material for: Population structure of Phanaeus vindex (Coleoptera: Scarabaeidae) in SE Michigan
Source: J Insect Sci. 2023 Jul 3;23(4):2. doi: 10.1093/jisesa/iead050 (PMC10317053; doi:10.1093/jisesa/iead050)
Supplement: iead050_suppl_Supplementary_Material [file iead050_suppl_supplementary_material.docx]

**Appendix S1**

Thomas Wassmer and Elise Armstrong

Population Structure of the Rainbow Scarab *Phanaeus vindex* (MacLeay 1819) in SE-Michigan


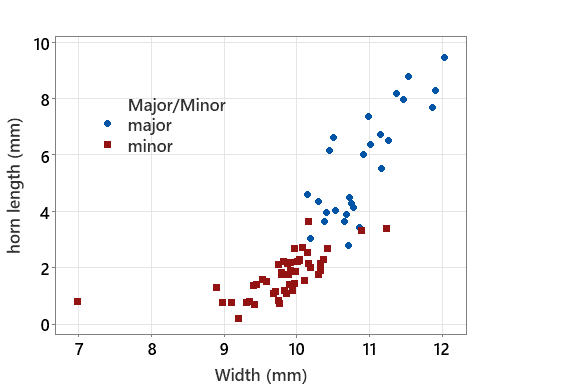


Figure S1: Scatterplot of horn length vs. width for 458 rainbow scarabs from 2019-2021 showing a curvilinear to sigmoid allometry.


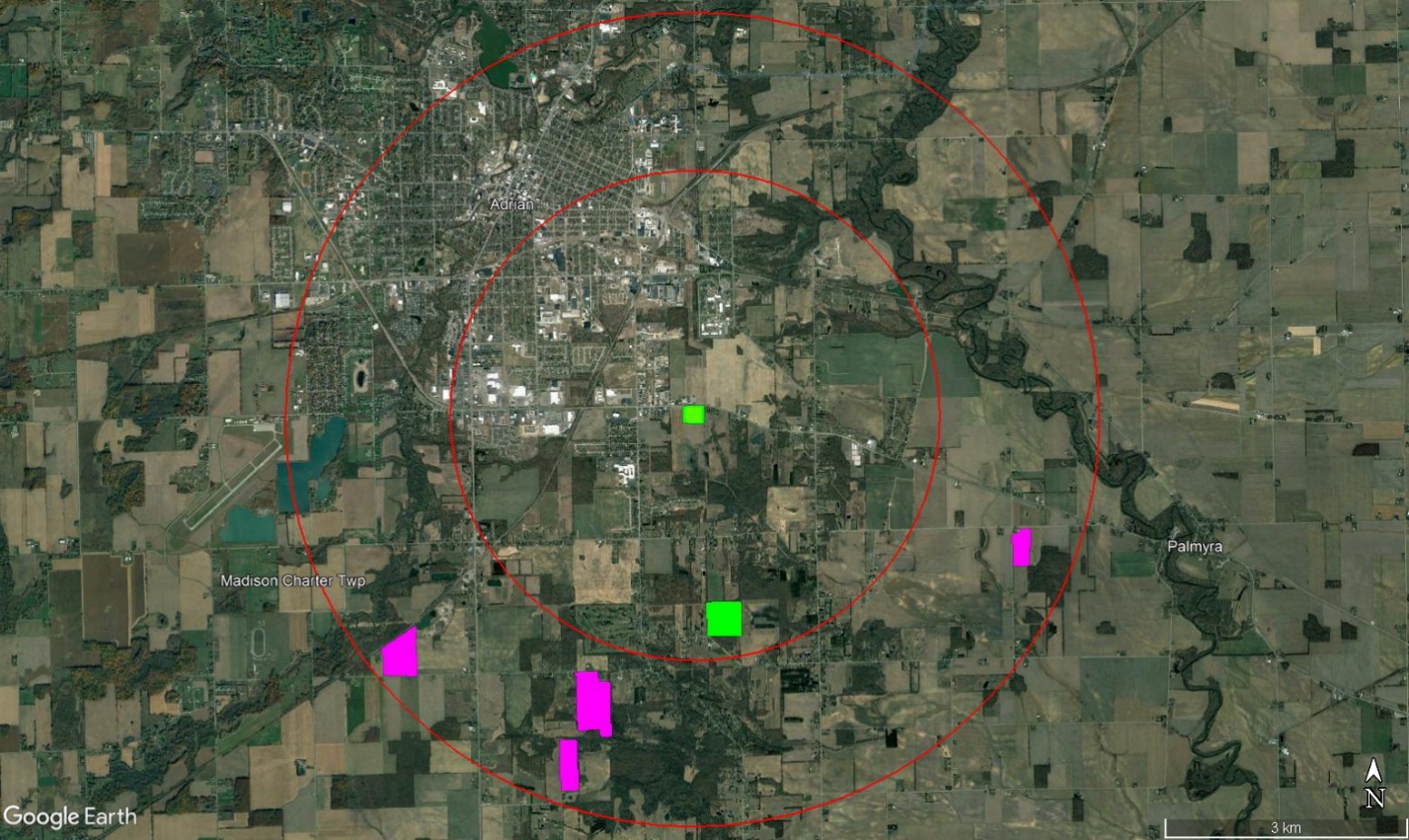


Figure S2: Locations of the two study sites, Carpenter Farm (green rectangle in center) and Deline Farm (green rectangle on bottom) within the local landscape mosaic of 5 by 7 km. The concentric circles denominate 3 and 5 km distance around Carpenter Farm. There are 4 additional farms in the outermost concentric segment (pink).
